# Effects of Surface Modification and Bulk Geometry on the Biotribological Behavior of Cross-Linked Polyethylene: Wear Testing and Finite Element Analysis

**DOI:** 10.1155/2015/435432

**Published:** 2015-10-25

**Authors:** Kenichi Watanabe, Masayuki Kyomoto, Kenichi Saiga, Shuji Taketomi, Hiroshi Inui, Yuho Kadono, Yoshio Takatori, Sakae Tanaka, Kazuhiko Ishihara, Toru Moro

**Affiliations:** ^1^Research Department, KYOCERA Medical Corporation, Osaka, Japan; ^2^Division of Science for Joint Reconstruction, Graduate School of Medicine, The University of Tokyo, Tokyo, Japan; ^3^Department of Materials Engineering, School of Engineering, The University of Tokyo, Tokyo, Japan; ^4^Orthopaedic Surgery, Sensory and Motor System Medicine, Surgical Sciences, Graduate School of Medicine, The University of Tokyo, Tokyo, Japan

## Abstract

The wear and creep deformation resistances of polymeric orthopedic bearing materials are both important for extending their longevity. In this study, we evaluated the wear and creep deformation resistances, including backside damage, of different polyethylene (PE) materials, namely, conventional PE, cross-linked PE (CLPE), and poly(2-methacryloyloxyethyl phosphorylcholine)- (PMPC-) grafted CLPE, through wear tests and finite element analysis. The gravimetric and volumetric degrees of wear of disks (3 or 6 mm in thickness) of these materials against a cobalt-chromium-molybdenum alloy pin were examined using a multidirectional pin-on-disk tester. Cross-linking and PMPC grafting decreased the gravimetric wear of the PE disks significantly. The volumetric wear at the bearing surface and the volumetric penetration in the backside of the 3-mm thick PE disk were higher than those of the 6-mm thick PE disk, regardless of the bearing material. The geometrical changes induced in the PE disks consisted of creep, because the calculated internal von Mises stress at the bearing side of all disks and that at the backside of the 3-mm thick disks exceeded their actual yield strengths. A highly hydrated bearing surface layer, formed by PMPC grafting, and a cross-linking-strengthened substrate of adequate thickness are essential for increasing the wear and creep deformation resistances.

## 1. Introduction

Ultra-High-molecular-weight polyethylene (UHMWPE; PE) has conventionally been used for orthopedic bearing materials since the 1960s. In addition, highly cross-linked PE (CLPE) is being used since the 1990s [1]. The degree of wear is one of the important indicators of the clinical performance of the bearing materials used for artificial joints, regardless of whether CLPE is used [2]. The wear particles generated from the acetabular liner used in total hip arthroplasty (THA) induce bone resorption and lead to aseptic loosening [3]. Hence, various strategies have been employed for reducing the number of PE wear particles generated and extending the longevity of polymeric orthopedic bearing materials [4]. To reduce the degree of PE wear and suppress bone resorption, we had previously developed a surface modification technology that involves synthetic phospholipid-polymer poly(2-methacryloyloxyethyl phosphorylcholine [MPC]) (PMPC) grafting. This technology results in increases in the lifetime of the acetabular liners [5, 6]. The polymer MPC is a very common lubricious, hydrophilic, and biologically inert one and is thus suitable for clinical use [7]. The modification of the surface of CLPE with a nanometer-scale layer of PMPC increases the lubricity of CLPE to the same level as that of articular cartilage under physiological conditions [5]. The grafting of PMPC onto acetabular liners resulted in a drastic reduction in CLPE wear during a long-term hip simulator test [8]; it also yielded good short-term results during clinical tests [9].

Creep deformation resistance is also an important indicator of the clinical performance of acetabular liners [10]. The geometry of the PE part is one of the factors that determine its creep deformation resistance [11, 12]. The dislocation caused by the impingement of the femoral stem neck and the acetabular liner is a common cause of early failure of THAs [13]. A large-diameter femoral head not only allows for an increase in the head/neck ratio, which is directly related to the range of motion prior to the impingement of the femoral stem neck and the acetabular liner, but also increases the jump distance [14]. Hence, large-diameter femoral heads have recently come to be used extensively for improving the stability of bearing surfaces [14, 15]. However, large-diameter femoral heads need to be used with thin acetabular liners, in order to ensure that the acetabular bone is retained, as not doing so can produce higher contact stresses, accelerating the wear and/or fracture of the acetabular liner [16].

Similarly, backside wear as well as the damage caused by volumetric penetration and circular scratching is also a serious problem with respect to the PE acetabular liners used in cementless THA and the PE inserts used in total knee arthroplasty (TKA) [17, 18]. High internal stress occurs in thin PE acetabular liners, and plastic flow is caused by the rubbing of the backside surface against the edge of the screw hole in the metal acetabular shell. A previous study reported that this internal stress as well as the plastic flow of PE increased with a decrease in the thickness of the PE bulk [19]. In contrast, another study reported that the wear of PE and CLPE acetabular liners increases significantly with an increase in the liner thickness [16]. It is therefore thought that the thickness of the PE bulk affects both the wear resistance and the creep deformation resistance. In other words, when evaluating the wear and creep deformation resistances of a polymeric material, the thickness of the test specimen must be taken into account.

The development of new biomaterials requires practical and economical screening methods for identifying the best-suited bearing materials before performing expensive joint simulator tests. A number of wear tests, which are performed under different conditions, have been developed such that they take into account the wear and fatigue mechanisms corresponding to clinical use [20, 21]. The ASTM F732-00 standard has been designed to evaluate bearing couples on the basis of the degree of polymer wear by using a pin-on-disk tester [22]. It also defines a method in which a disk-shaped polymer specimen is loaded with a hemispherical cobalt-chromium-molybdenum (Co-Cr-Mo) alloy pin. If one were to employ this method while using a plate with a screw hole as the backplate of the disk specimen, one could evaluate the damage undergone by the backside of the disk without having to perform an expensive joint simulator test [23]. In addition, this method is suitable for evaluating hydrated polymers because a lubricant is applied on the bearing surface during every loading cycle [24].

The purpose of this study was to evaluate the wear and creep deformation resistances, including the extent of backside damage, of various PE materials using test specimens 3 and 6 mm in thickness. We sought to answer four questions: will the choice of the PE material used affect the (1) wear resistance and (2) creep deformation resistance of the test specimens? Further, will the thickness of the PE test specimens affect their (3) wear resistance and (4) creep deformation resistance?

## 2. Materials and Methods

### 2.1. Bearing Materials and PMPC Grafting

Compression-molded bars of PE (GUR1020 resin; Quadrant PHS Deutschland GmbH, Vreden, Germany) were prepared. This material is referred to as untreated PE. A few bars were irradiated with a 50-kGy dose of gamma rays in N_2_ gas and annealed at 120°C for 7.5 h in N_2_ gas to facilitate cross-linking. This material is referred to as untreated CLPE. PMPC was grafted onto the surfaces of the CLPE samples through photoinduced graft polymerization [5, 6]. Polymerization was performed on the surfaces of the untreated CLPE samples by irradiating them with ultraviolet (UV) radiation with an intensity of 5 mW/cm^2^ at 60°C for 90 min in a 0.5 mol/L aqueous solution of MPC (NOF Corp., Tokyo, Japan). Hereafter, this material is referred to as PMPC-grafted CLPE. The samples were then sterilized using gamma rays at a dose of 25 kGy in N_2_ gas.

### 2.2. Cross-Link Density

The cross-link densities of the materials were evaluated using a previously reported method [5, 25]. The specimens (23 × 23 × 1 mm) were weighed (approximately 0.5 g), allowed to swell for 72 h in* p*-xylene containing 0.5 mass% 2-*t*-butyl-4-methylphenol at 130°C, and then reweighed. The samples were then immersed in acetone, dried at 60°C under vacuum, and reweighed. The swelling ratio was determined from the increase in the weights of the samples and densities of the PE used and xylene. The network chain density was calculated using the Flory-Rehner equation [26]. The cross-link density was defined as the mole fraction of the cross-linked units.

### 2.3. Wettability Test

The contact angle of a static droplet of water on specimens (100 × 10 × 3 mm) of each material was measured using the sessile drop method; an optical bench-type contact angle goniometer (Model DM300; Kyowa Interface Science, Saitama, Japan) was employed for the purpose. Drops of purified water (1 *μ*L) were deposited on the specimens, and the contact angles were measured directly after 60 s using a microscope. Fifteen areas were evaluated on each sample, and the mean values and the standard deviation were calculated.

### 2.4. Pin-on-Disk Test

**Figure 1:**
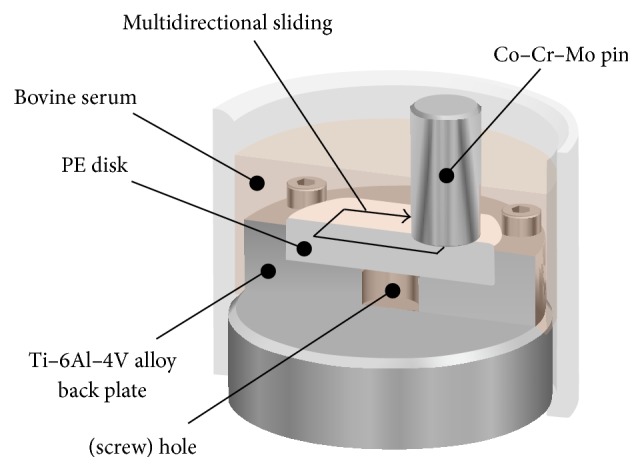
Schematic illustration of the multidirectional pin-on-disk test.

The wear and creep deformation resistances of the bearing materials were examined using a pin-on-disk tester (Ortho POD; AMTI, Watertown, MA, USA), in keeping with the ASTM F732-00 standard. The various PE materials were used to fabricate disks with a thickness of 3 or 6 mm, while a Co-Cr-Mo alloy was used for the counter hemispherical pin (Figure 1). The pin had a surface curvature radius of 30 mm and a surface roughness, *R*_*a*_, of less than 0.01 *μ*m. Its backplate was made of a titanium alloy and had a sham screw hole with a diameter of 8 mm in the center. A mixture of 27 vol% fetal bovine serum (Biowest, Nuaillé, France), 20 mM/L ethylene diamine-N,N,N′,N′-tetraacetic acid (EDTA), and 0.1 mass% sodium azide was used at 37°C as the lubricant.

Multidirectional pin-on-disk tests were performed; the motion was in the shape of a rectangle with dimensions of 5 × 10 mm. The test conditions were the following: static load of 213 N, motion speed of 30 mm/s, and maximum test duration of 1.0 × 10^6^ cycles. The disks were weighed every 0.25 × 10^6^ cycles to evaluate their gravimetric wear, in keeping with the ISO 14242-2 standard [27]. The lubricant was also replaced every 0.25 × 10^6^ cycles. Soak controls were used to compensate for fluid absorption by the specimens of the same group. Because the gravimetric method was used, the decreases in the weights of the tested specimens were corrected for by subtracting the weight gain resulting from the use of the soak control. The volume of wear was calculated by dividing the individual weight of the disks by the density of the corresponding material: 0.937 g/mm^3^ for untreated CLPE, 0.941 g/mm^3^ for untreated CLPE, and 0.941 g/mm^3^ for PMPC-grafted CLPE. The wear rate was calculated with the least squares linear regression method while using data corresponding to points other than the zero-time point. After the completion of the multidirectional pin-on-disk test, the wear of the bearing surface and the depth of penetration in the backside surface of all the disks were measured using a noncontact optical three-dimensional (3D) profiler (Talysurf CCI Lite; Taylor Hobson Ltd., Leicester, UK).

### 2.5. Finite Element Analysis

**Figure 2:**
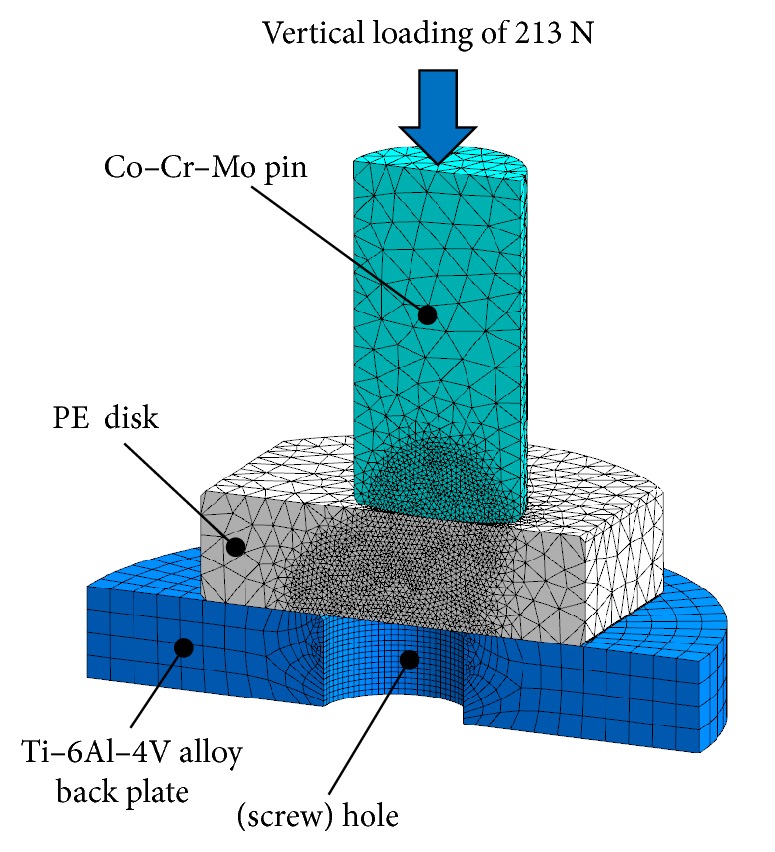
Schematic illustration of FEA used to determine the internal stress in the PE disks during the pin-on-disk test.

To estimate the internal stress in the PE disks, finite element analysis (FEA) was performed using an FEA software program (ANSYS 14.5; Ansys, Inc., Canonsburg, PA, USA). A computer-aided 3D model of the pin-on-disk test conditions was created using a computer-aided design (CAD) software program (Solid Edge ST3; Siemens PLM Software, Plano, TX, USA). This model was then imported into the FEA program. A static vertical load of 213 N was applied to the disks through the pin (Figure 2). With respect to the physical properties of the different PE materials, Young's moduli of the untreated PE, untreated CLPE, and PMPC-grafted CLPE were taken to be 1.06, 1.05, and 1.10 GPa, respectively, while their Poisson's ratios were assumed to be 0.41, 0.46, and 0.43, respectively.

### 2.6. Statistical Analysis

The mean values for the three material groups (untreated PE, untreated CLPE, and PMPC-grafted CLPE) were compared using one-factor analysis of variance (ANOVA). The significant differences between the comparable properties were determined through post hoc testing using Tukey-Kramer's method. The mean values of the two thickness groups (3 and 6 mm) for each material were compared by using Student's *t*-test. All statistical analyses were performed using an add-on (Statcel 3; OMS Publishing, Tokorozawa, Japan) for Microsoft Excel 2007 (Microsoft Corp., Redmond, WA, USA).

## 3. Results

**Figure 3:**
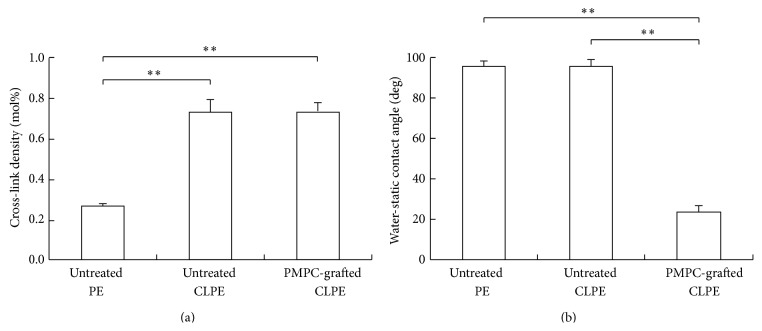
(a) Cross-link densities and (b) static water contact angles of untreated PE, untreated CLPE, and PMPC-grafted CLPE. The bar indicates the standard deviation. *∗∗* indicates *p* < 0.01 as per Tukey-Kramer's test.

The cross-link densities of untreated CLPE and PMPC-grafted CLPE were significantly higher (*p* < 0.01) than that of untreated PE (Figure 3(a)). The static water contact angle of PMPC-grafted CLPE was significantly smaller (*p* < 0.01) than those of untreated PE and CLPE (Figure 3(b)).

**Figure 4:**
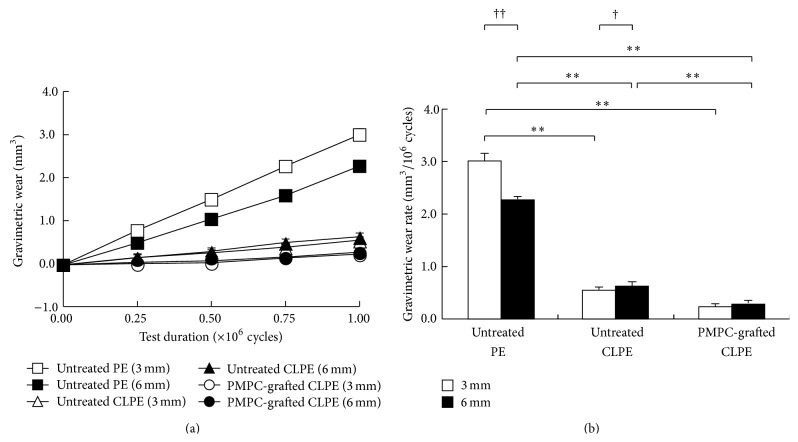
(a) Degrees of gravimetric wear and (b) gravimetric wear rates of untreated PE, untreated CLPE, and PMPC-grafted CLPE, determined using disks 3 and 6 mm in thickness. The bar indicates the standard deviation. *∗∗* indicates *p* < 0.01 as per Tukey-Kramer's test and † indicates *p* < 0.05 and †† indicates *p* < 0.01 as per Student's *t*-test.

The gravimetric wear rates for all disks after the end of the pin-on-disk test are shown in Figure 4(a). For the 3-mm thick disk group, the gravimetric wear rates of untreated CLPE and PMPC-grafted CLPE disks were significantly lower (*p* < 0.01) than that of the untreated PE disks; however, the difference between the untreated CLPE and PMPC-grafted CLPE disks was not significant (Figure 4(b)). For the 6-mm thick disk group, the wear rates of untreated CLPE and PMPC-grafted CLPE disks were significantly lower (*p* < 0.01) than that of the untreated PE disks. Furthermore, the wear rate of PMPC-grafted CLPE was significantly lower (*p* < 0.01) than that of untreated CLPE. The wear rate of the 3-mm thick untreated PE disks was significantly larger than that of the 6-mm thick disks (*p* < 0.01). On the other hand, the wear rate of the 3-mm thick untreated CLPE disks was significantly lower than that of the 6-mm thick disks (*p* < 0.05). In the case of PMPC-grafted CLPE, there were no significant differences in the wear rates of the 3 and 6 mm disks.

**Figure 5:**
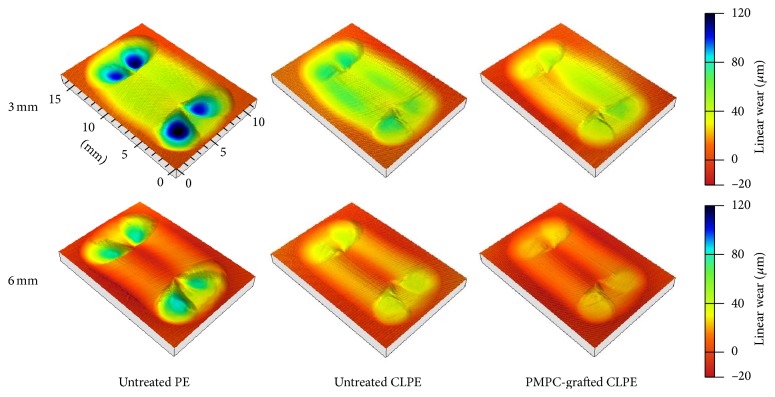
Degree of volumetric wear of the bearing surfaces of the untreated PE, untreated CLPE, and PMPC-grafted CLPE disks 3 and 6 mm in thickness.

**Figure 6:**
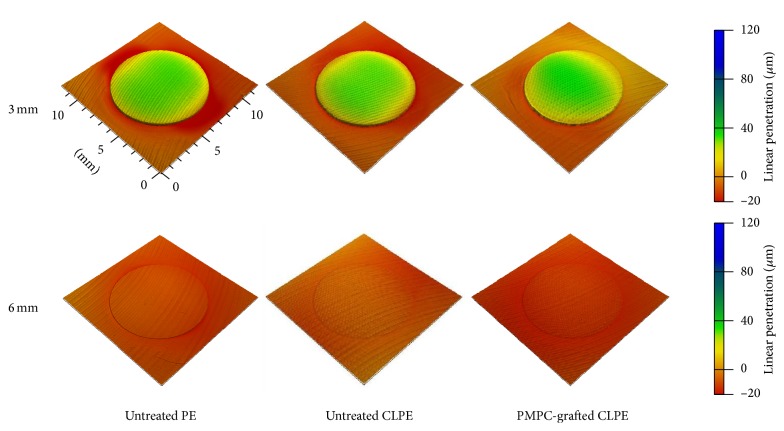
Volumetric penetration of the backside surfaces of the untreated PE, untreated CLPE, and PMPC-grafted CLPE disks 3 and 6 mm in thickness.

**Figure 7:**
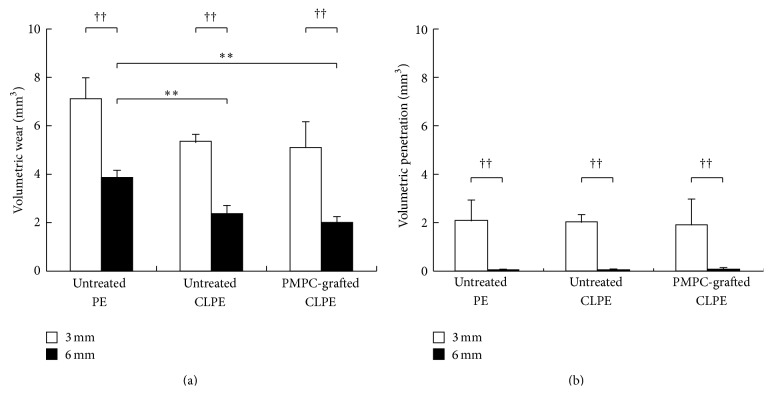
(a) Volumetric wears of the bearing surfaces and (b) volumetric penetration in the backside surfaces of the untreated PE, untreated CLPE, and PMPC-grafted CLPE disks. The bar indicates the standard deviation. *∗∗* indicates *p* < 0.01 as per Tukey-Kramer's test and †† indicates *p* < 0.01 as per Student's *t*-test.

The 3D profile of the disks showed that all the disks exhibited substantial volumetric wear of the bearing surface, with the backside surface penetrating into the sham screw hole (Figures 5 and 6). The differences in the degrees of volumetric wear for the 3-mm thick disks of the different materials were not significant (Figure 7(a)). On the other hand, the volumetric wear of the 6-mm thick disk of untreated PE was significantly larger (*p* < 0.01) than those of the disks of untreated CLPE and PMPC-grafted CLPE. For each material group, the volumetric wear of the 3-mm thick disks was significantly larger (*p* < 0.01) than that of the 6-mm thick disks. The differences in the volumetric penetration for both the 3-mm thick and 6-mm thick disks of the various materials were not significant. For each material group, the volumetric penetration of the 3-mm thick disks was significantly greater (*p* < 0.01) than that of the 6-mm thick disks (Figure 7(b)).

**Figure 8:**
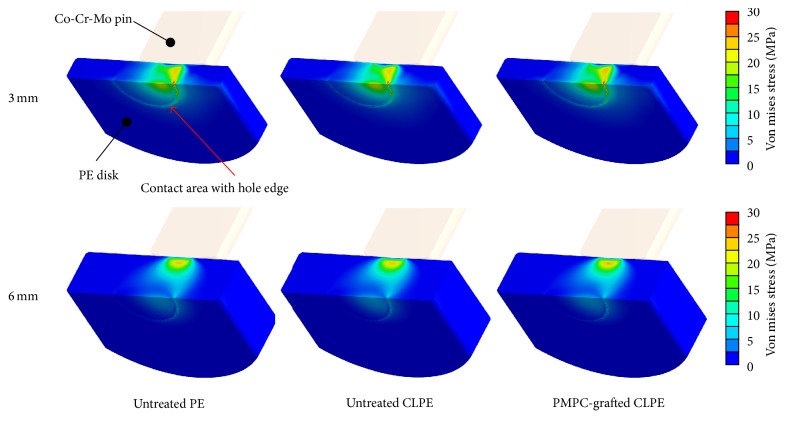
von Mises stress distributions of the untreated PE, untreated CLPE, and PMPC-grafted CLPE disks.

The von Mises stress distribution for all the disks was estimated using FEA (Figure 8). The internal von Mises stress at the bearing side was almost the same for all the materials, as shown in Table 1. Although the stress at the backside was almost the same for all the material groups for both the 3-mm thick and the 6-mm thick disks, the stress in the 3-mm thick disks was computationally much higher than that in the 6-mm thick disks. Further, it exceeded their respective actual yield strengths by a wide margin.

## 4. Discussion

In this study, we evaluated the wear and creep deformation resistances, including the degree of backside damage, of conventional PE, CLPE, and PMPC-grafted CLPE using disks with thicknesses of 3 and 6 mm. We attempted to answer the following four questions: does the choice of the PE material affect the (1) wear resistance and (2) creep deformation resistance of the test specimens? In addition, will the thickness of the PE specimens affect their (3) wear resistance and (4) creep deformation resistance?

Cross-linking decreased the gravimetric wear of PE significantly, while PMPC grafting decreased it even further. It is known that cross-linking can improve the wear resistance of the acetabular liners used in artificial hip joints [28, 29]. PMPC-grafted CLPE has also been shown to exhibit lower wear than that seen for CLPE [5, 8]; it is thought that the decrease in wear is caused by a surface hydration lubrication mechanism [30]. According to a previous study, the hydrated PMPC layer has a clear effect on the friction response of the polymer; the dynamic coefficient of friction of PMPC-grafted CLPE is significantly lower than that of untreated CLPE [5]. This is attributable to the fluid film formed by the hydrated PMPC layer on the CLPE surface [5]. In the case of natural synovial joints, lubrication is provided by a hydrated layer that consists of chondrocytes; the surrounding matrix macromolecules (e.g., proteoglycans, glycosaminoglycans, and collagens) are also essential for lubrication, while a surface layer of active phospholipids (e.g., phosphatidylcholine derivatives) that covers the surface of the joint cartilage provides hydrophilicity and works as an effective boundary lubricant [31, 32]. Hence, a phospholipid-like layer grafted on the bearing surface of artificial joints may afford ideal hydrophilicity and lubricity, that is, similar to those of physiological joint surfaces.

Even though gravimetric evaluations were performed, measuring the volumetric wear of the bearing surface using an optical 3D profiler did not allow for the detection of significant differences between the three PE material groups in the case of the 3-mm thick disks and between untreated CLPE and PMPC-grafted CLPE in the case of the 6-mm thick disks. The internal von Mises stress at the bearing side for both the 3-mm thick and the 6-mm thick disks reached approximately 23 MPa, which is reportedly the actual yield strength of PE materials [33, 34]. We therefore assumed that the volumetric wear measured in this study consisted of not only the true wear but also the creep deformation of the PE substrate. Since the volume of creep deformation was significantly greater than that of true wear, the differences in the volumetric wear among the three materials for the 3-mm thick disks as well as that between untreated CLPE and PMPC-grafted CLPE for the 6-mm thick disks (which exhibited large creep deformation) might not be significant.

The modification processes investigated, namely, cross-linking and PMPC grafting, did not affect the creep deformation resistance (Figure 7(b)). It has been reported that creep deformation decreases with an increase in the irradiation dose [35]. It was assumed that the effects of an irradiation dose of 50 kGy on creep deformation would be too small to allow for differences in the creep deformations of the PE and CLPE samples to be detected. In the case of PMPC grafting, previous studies have indicated that photoinduced graft polymerization does not affect the mechanical properties of the CLPE substrate [5, 33]. In this study, we used disks 3 or 6 mm in thickness. Thus, the backside surface, where the maximum stress appeared in the case of the 3-mm thick disks (Figure 8), might not be affected by UV irradiation either. Shyichuk et al. reported that UV irradiation affects the mechanical properties of PE, especially on the submillimeter scale [36]. In the present study, UV irradiation was performed with an intensity of 5 mW/cm^2^ for 90 min; this resulted in an energy of 0.3 × 10^6^ J/m^2^, which was low compared to that used by Shyichuk et al. (3.6 × 10^6^ J/m^2^ for 3 weeks) [36].

Although disk thickness did affect the gravimetric wear rate in the case of the untreated PE and untreated CLPE disks, the effects were not the same. For untreated PE, the gravimetric wear rate increased with a decrease in the thickness of the disk specimens. We assumed that this was attributable to an increase in the contact area, owing to the creep deformation of the bearing surface (Figure 5). For untreated CLPE, in contrast, the gravimetric wear rate increased with an increase in the thickness of the disk specimen. Shen et al. reported that, in an* in vitro* study, the wear rate of a CLPE acetabular liner increased with an increase in the liner thickness [16]. In the present study, the losses in the weights of the untreated CLPE disks (0.043 and −0.070 mg for the 3 and 6-mm thick PE disks, resp.) were much smaller than the increases in their weights owing to fluid absorption (0.487 and 0.673 mg for the 3 and 6-mm thick PE disks, resp.). Thus, these phenomena may not be detectable using the gravimetric method.

The volumetric penetration in the backside surfaces increased with a decrease in disk thickness. The internal von Mises stress in all the 3-mm thick disks was more than 28 MPa in the backside against the edge of the sham screw hole; this was greater than the actual yield strength of the PE materials (Table 1). It is likely that the fact that the internal stress was greater than the actual yield strength is what resulted in the large degree of volumetric penetration. On the other hand, the von Mises stress in all the 6-mm thick disks was less than 8 MPa. This internal stress did not lead to changes in the geometry of the PE disks. However, it affected not only the backside surface but also the bearing surface of the disks. The degree of volumetric wear, which consisted of true wear and creep deformation, was greater in the thinner (3 mm) disks (Figure 7(a)) for every material, regardless of the differences in the degrees of gravimetric wear (Figure 3) and the internal von Mises stress (Table 1). We assumed that the increase in the degree of volumetric wear in thinner (3 mm) disks was partially owing to the volumetric penetration in the backside surface. Oonishi et al. have also reported that the wear rate of PE cups in cemented THA increased with a decrease in the cup thickness [37].

Various wear tester designs and testing conditions have been employed to evaluate the suitability of bearing materials for clinical use [4]. The ASTM F732-00 standard provides the test parameters that are used as guidelines for producing wear rates similar to those observed* in vivo*. In this study, we used PE materials for the disks and a Co-Cr-Mo alloy for the pin, while referring to the Appendix  3 of ASTM F732-00 standard. Baykal et al. reviewed the wear rate data for PE materials obtained using various pin-on-disk testers [38]: the most reviewed studies used PE materials for the pin side and a metal for the disk side, in order to keep the contact area of the bearing interface constant. The test couple employed in the present study, which consisted of PE materials for the disk side and a Co-Cr-Mo alloy for the pin side, has a number of advantages. For example, one can evaluate the effects of the sample geometry (i.e., thickness), backside penetration in the presence and absence of a backplate hole, and the hydration lubrication mechanism, which involves dehydration/rehydration processes. However, it also has a disadvantage in that the contact area does not remain constant. There have been several studies on PE materials involving pin-on-disk tests in which the PE materials were used for the disks [39, 40]. Tomita et al. determined the effectiveness of adding vitamin E to PE for preventing fatigue crack by using metal pin-on-PE disk tests [39]. Saikko et al. developed a test system named Random POD. This system simulated a hip joint in the case of a bearing couple of a PE pin-on-Co-Cr-Mo alloy disk or simulated a knee joint in the case of a bearing couple of Co-Cr-Mo alloy pin-on-PE disk [40, 41]. While they simulated the conditions of knee joints, this bearing couple consisting of a PE disk against a Co-Cr-Mo alloy pin is suitable for simulating not only artificial knee joints, but also artificial hip joints. This is because we could evaluate bearing materials while taking into account sample geometry.

Finally, there were several limitations of the present study. To begin with, we used a small cycling period (1.0 × 10^6^ cycles) for the multidirectional wear test performed using the pin-on-disk tester as a preliminary examination, in keeping with the ASTM F732-00 standard. This duration may not be large enough to correctly simulate the loading and motion conditions associated with physical walking or the daily routine of patients. Nevertheless, we believe that the multidirectional wear test does provide some indication of the wear and fatigue performances of the PE materials [38]. In addition, we calculated the wear rate using data points other than those obtained at the zero-time point; thus, the steady state of the wear process was not known. It has been reported that there are two phases of wear, namely, the run-in state and the steady state, during wear tests [22, 42, 43]. In this study, the steady state of the wear process was not fully known because wear data after 1.0 × 10^6^ cycles is not available; however, the coefficient of determination ranged from 0.94 to 0.99. We assumed that the run-in state might be over after 0.25 × 10^6^ cycles [42]. However, we are in the process of conducting longer hip simulator studies using PMPC-grafted CLPE liners and have been able to confirm that the PMPC-grafted liners exhibit almost no wear after 20 × 10^6^ cycles [8]. Second, the conditions under which the pin-on-disk wear test, in which a disk-shaped polymer specimen is loaded with a hemispherical pin, increases is performed, the contact area increases owing to an increase in the volumetric wear. While this is a limitation, it probably reflects the actual clinical conditions. Third, during the FEA, we simulated static conditions and used only a vertical load. In actuality, the pin-on-disk test involves dynamic conditions, and the disk is subjected to not only vertical loads but also multidirectional kinetic forces. In addition, the FEA was a linear analysis, while creep deformation should be simulated using a nonlinear analysis [44].

## 5. Conclusions

The main conclusions of the present study can be listed as follows: (1) the cross-linking of PE improved its wear resistance, while PMPC grafting improved the resistance even further. (2) Cross-linking through irradiation at a dose of 50 kGy and PMPC grafting did not affect creep deformation. (3) An increase in disk thickness tended to decrease the gravimetric wear for untreated PE; however, the effect was too small to detect in the case of untreated and PMPC-grafted CLPE. (4) The thickness of the test samples had an effect on the creep deformation resistance; this was true for both the bearing and the backside surfaces.

A hydrated bearing surface and a bearing substrate of the appropriate thickness are essential for improving the wear and creep deformation resistances. We believe that PMPC-grafted CLPE is a promising bearing material for increasing the longevity of artificial joints. Further, the pin-on-disk test is a useful one for evaluating bearing biomaterials in a practical and economical manner.

## Figures and Tables

**Table 1:** Maximum von Mises stresses in the bearing and backside surfaces of the untreated PE, untreated CLPE, and PMPC-grafted CLPE disks.

Thickness (mm)	Maximum von Mises stress (MPa)					
	Bearing surface			Backside surface		
	Untreated PE	Untreated CLPE	PMPC-grafted CLPE	Untreated PE	Untreated CLPE	PMPC-grafted CLPE
3	23.2	23.4	23.9	29.0	28.5	29.0
6	22.7	22.6	23.3	7.3	7.3	7.3
